# Bilirubin and postpartum depression: an observational and Mendelian randomization study

**DOI:** 10.3389/fpsyt.2024.1277415

**Published:** 2024-03-08

**Authors:** Yi Liu, Zhihao Wang, Duo Li, Bin Lv

**Affiliations:** ^1^ Department of Gynecology and Obstetrics, West China Second University Hospital, Sichuan University, Key Laboratory of Birth Defects and Related Diseases of Women and Children (Sichuan University), Ministry of Education, Chengdu, China; ^2^ Department of Thoracic Surgery and Institute of Thoracic Oncology, West China Hospital, Sichuan University, Chengdu, China; ^3^ Department of Neurosurgery, West China Hospital, Sichuan University, Chengdu, China; ^4^ Department of Anesthesiology, West China Hospital, Sichuan University, Chengdu, China

**Keywords:** postpartum depression, serum bilirubin, risk factors, Mendelian randomization, oxidative stress

## Abstract

**Background:**

Postpartum depression (PPD) is one of the most common complications of delivery and is usually disregarded. Several risk factors of PPD have been identified, but its pathogenesis has not been completely understood. Serum bilirubin has been found to be a predictor of depression, whose relationship with PPD has not been investigated.

**Methods:**

Observational research was performed followed by a two-sample Mendelian randomization (MR) analysis. From 2017 to 2020, the clinical data of pregnant women were retrospectively extracted. Logistic regression and random forest algorithm were employed to assess the risk factors of PPD, including the serum levels of total bilirubin and direct bilirubin. To further explore their potential causality, univariable and multivariable Mendelian randomization (MVMR) were conducted. Sensitivity analyses for MR were performed to test the robustness of causal inference.

**Results:**

A total of 1,810 patients were included in the PPD cohort, of which 631 (34.87%) were diagnosed with PPD. Compared with the control group, PPD patients had a significantly lower level of total bilirubin (9.2 μmol/L, IQR 7.7, 11.0 in PPD; 9.7 μmol/L, IQR 8.0, 12.0 in control, *P* < 0.001) and direct bilirubin (2.0 μmol/L, IQR 1.6, 2.6 in PPD; 2.2 μmol/L, IQR 1.7, 2.9 in control, *P* < 0.003). The prediction model identified eight independent predictive factors of PPD, in which elevated total bilirubin served as a protective factor (OR = 0.94, 95% CI 0.90–0.99, *P* = 0.024). In the MR analyses, genetically predicted total bilirubin was associated with decreased risk of PPD (IVW: OR = 0.86, 95% CI 0.76–0.97, *P* = 0.006), which remained consistent after adjusting educational attainment, income, and gestational diabetes mellitus. Conversely, there is a lack of solid evidence to support the causal relationship between PPD and bilirubin.

**Conclusion:**

Our results suggested that decreased total bilirubin was associated with the incidence of PPD. Future studies are warranted to investigate its potential mechanisms and illuminate the pathogenesis of PPD.

## 1 Introduction

Postpartum depression (PPD) mainly manifests as a major depressive episode combined with multiple mental and physical symptoms during the postpartum period (1). The prevalence of PPD, which has been underestimated previously, varies from 3% to 38% in different nations and is usually higher in developing countries (2, 3). Apart from the mothers, their partners and offspring may also suffer from PPD. It was estimated that the incidence of paternal depression was approximately 8% (4). For infants, retardation of weight and height was observed, as well as a decrease in cognitive and emotional development (5). The recommended multidisciplinary management of PPD includes psychosocial, psychological, pharmacological, and somatic interventions, whose effectiveness needs to be improved (6, 7). The pathogenesis of PPD has not been fully understood, and the known risk factors could be summarized into several aspects: medical history of primary mood disorders such as anxiety (8); sociodemographic characteristics such as education, age, and income (3, 9, 10); biological status during the perinatal period such as thyroid function (11); and obstetrics-related factors such as mode of delivery and preterm birth (12, 13). Given the heavy burden of the disease and the huge barrier to treatment, further studies are desperately warranted.

Bilirubin, which is the end product of heme catabolism, is cytotoxic to the central nervous system at high concentrations, while it also serves as an antioxidant at low concentrations in the serum (14). Bilirubin was found to be involved in many chronic diseases, including cardiovascular diseases, diabetes, neuropsychiatric diseases, and certain cancers (15). However, the relationship between bilirubin and depression is still controversial. It has been reported that the high level of serum total bilirubin is related to an increased risk of poststroke depression in observational studies (16, 17). In patients with diabetes, a higher level of indirect bilirubin was found in those with depression (18). On the other hand, a small-sample study confirmed a lower nocturnal bilirubin level in winter seasonal patients (19). Total bilirubin was a protective biomarker of depression in data mining from the National Health and Nutrition Examination Survey (NHANES) (20). Currently, no study has focused on the relationship between the serum bilirubin level and PPD.

The two-sample Mendelian randomization (MR) is an excellent tool for clinicians to investigate causality between exposure factors and outcomes. During the process, single nucleotide polymorphisms (SNPs) from genome-wide association studies (GWASs) are identified as instrumental variables (IVs) (21). To achieve accurate causal inference, MR analysis must satisfy the following three assumptions: 1) in univariable MR, the IVs are associated with exposure. In multivariable MR (MVMR), they are associated with at least one of the exposures; 2) IVs are independent of all potential confounders; and 3) IVs are assumed to be independent of the outcome (22). Compared with observational studies, an MR study has a better performance in controlling confounding and reverse causation (23). By applying MR analysis, researchers have tried to investigate the relationship between omega-3 fatty acids and perinatal depression (24). In another MR study, major depressive disorder was reported to be significantly associated with decreased bilirubin (25). With the continuous construction of GWAS, it is now possible to investigate PPD using the MR approach.

In the present study, we combined independent clinical analysis and two-sample MR analysis, intended to shed light on the association and causality between bilirubin and PPD for the first time and provide solid evidence of early monitoring and intervention of PPD.

## 2 Materials and methods

### 2.1 Study design and data sources

**Figure 1:**
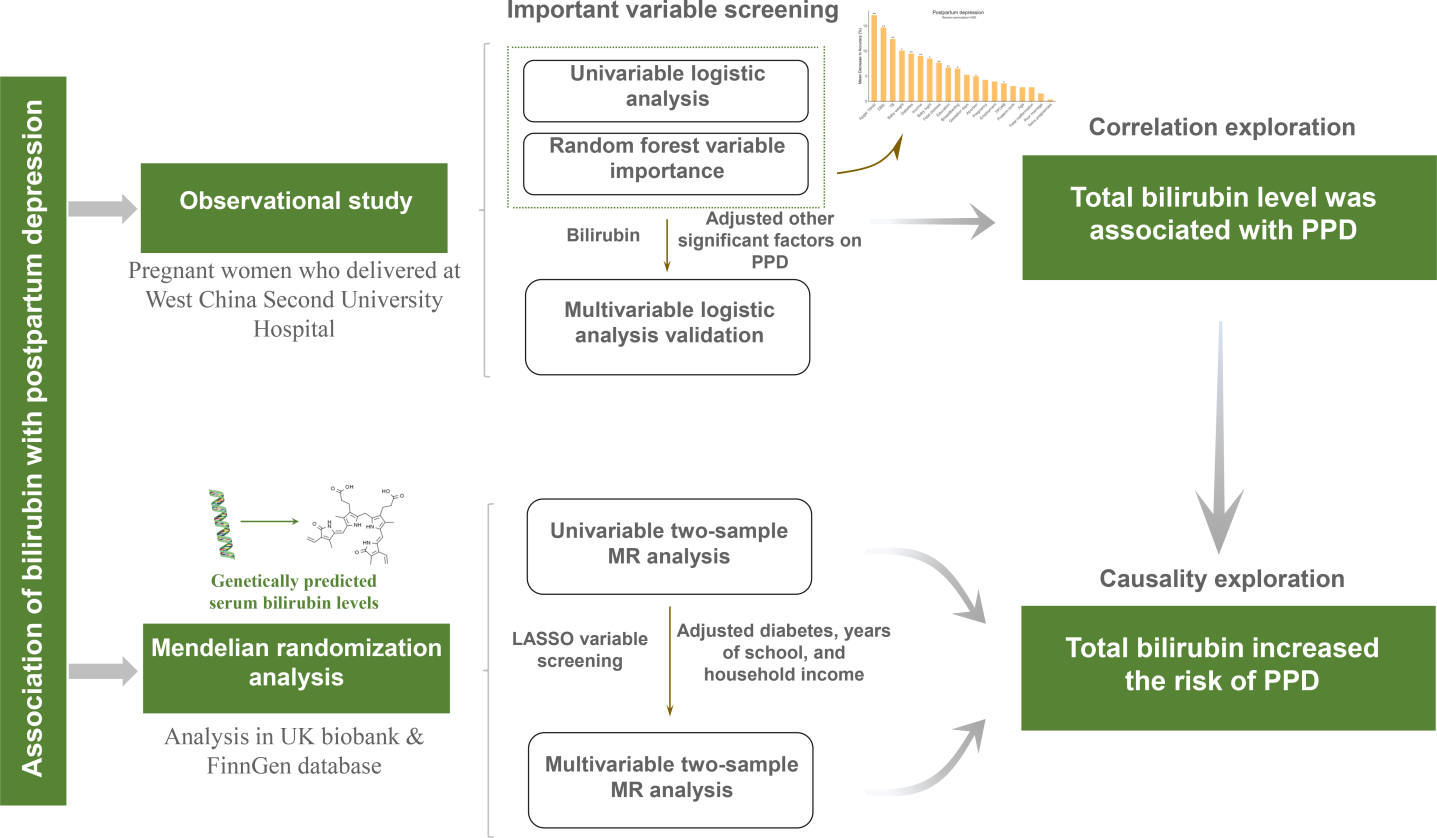
Flowchart of the study design. PPD, postpartum depression; MR, Mendelian randomization; UK, United Kingdom; LASSO, least absolute shrinkage and selection operator.

This study is composed of two major sections which are summarized in **Figure 1**. First, a cohort of PPD was built and important variables including total bilirubin and direct bilirubin were screened and validated to determine whether serum bilirubin level is associated with the incidence of PPD. Second, a two-sample MR analysis was performed to further investigate the causality between bilirubin and PPD with GWAS summary statistics.

For the observational study, pregnant women whose perinatal examinations and delivery were conducted at West China Second University Hospital, Sichuan University from 2017 to 2020 were selected for the present study. Participants were screened for eligibility. The inclusion criteria were as follows: a) participants who underwent regular examinations and delivered at West China Second University Hospital, Sichuan University; b) participants with a gestational age of ≥28 weeks; and c) participants who gave consent to participate and be followed up. The exclusion criteria were a) pre-existing mental illness, b) intellectual disability, and c) communication disorders.

All participants were followed up until 1 year after delivery.

### 2.2 Data process of the observational studies

The data of 62 variables were collected. Demographic information was collected by the electronic medical record system of West China Second University Hospital, Sichuan University. Social information was collected through questionnaires during the late stages of pregnancy (after 28 weeks until before delivery). The assessment of social support was carried out using the Social Support Rating Scale (SSRS), a well-established tool that has demonstrated high reliability and validity within the Chinese population (26). The SSRS consists of 10 items across three domains: objective support, subjective support, and utilization of social support. A score of 35 or less indicated low social support (27). Clinical characteristics were assessed and documented by eligible clinicians. Relevant laboratory indicators were extracted from the laboratory information system of West China Second University Hospital, Sichuan University during the late stages of pregnancy. Specifically, total bilirubin was measured via the vanadate oxidation method by Total Bilirubin_2 Reagents (Siemens Healthcare Diagnostics Inc., USA), with a reference value of 5–23 μmol/L for adolescents and adults. To evaluate the depression state of each participant, the Edinburgh Postnatal Depression Scale (EPDS) was used at 3 months postpartum (28). Participants who scored 13 or more were regarded as PPD (29).

To control the quality of the questionnaires, an interview for every participant was conducted by trained investigators in separate rooms. During the interviews, the confidentiality of this investigation was declared and the authenticity of the questionnaire was emphasized. Once the questionnaires were completed, all the items were checked by the investigators.

### 2.3 Two-sample MR analysis

#### 2.3.1 GWAS for bilirubin

GWASs for total circulating bilirubin (357,198 individuals) and direct bilirubin (418,830 individuals) were extracted from the UK Biobank database, with the raw data adjusted for covariates such as age, sex, sociodemographic features, and recruitment center of the participants as well as potential technical confounders including sampling time, fasting time, and sample dilution factor (30). The UK Biobank is a large-scale, long-term prospective cohort study that recruited approximately 500,000 individuals aged between 40 and 69 years from across Great Britain between 2006 and 2010 (31). Both total bilirubin and direct bilirubin were measured by a colorimetric assay (Beckman Coulter United Kingdom Ltd., Beckman Coulter AU5800 analyzer) (32).

#### 2.3.2 GWAS for potential confounders

To avoid the interference of potential confounders, genetic instruments of education, income, and gestational diabetes mellitus were obtained from the largest available studies. SNPs for educational attainment were collected from the GWAS dataset from the Social Science Genetic Association Consortium (SSGAC) with 766,345 individuals (33). As a result of the heterogeneity of educational systems in different regions and cultures, the completed number of schooling years was used to represent educational attainment based on the 1997 International Standard Classification of Education (ISCED) of the United Nations Educational, Scientific and Cultural Organization (34).

GWAS for the average total household income before tax (397,751 individuals) from the Medical Research Council Integrative Epidemiology Unit (MRC-IEU) consortium was applied, which was derived from the UK Biobank database by PHESANT (35). The average total household income before tax measured as a grade variable was self-reported by the UK Biobank participants voluntarily.

GWAS for gestational diabetes mellitus (9,837 cases, 162,622 individuals) was obtained from the FinnGen consortium (R7 data release) (https://www.finngen.fi/en). The FinnGen study is a nationwide cohort that combined genome information with digital medical data of participants who were over 18 years of age and lived in Finland (36). Gestational diabetes mellitus was defined as O244 in the 10th edition of the International Classification of Diseases criteria. In practice, the oral glucose tolerance test was recommended during 24–28 weeks of gestation. An additional test was recommended for high-risk women between 12 and 16 weeks of pregnancy. Participants with any abnormal venous plasma glucose result (fasting plasma glucose ≥5.3 mmol/L, 1-h glucose ≥10.0 mmol/L or 2-h glucose ≥8.6 mmol/L) in a single glucose tolerance test were diagnosed as having gestational diabetes (37).

#### 2.3.3 GWAS for postpartum depression

GWAS for PPD (13,657 cases, 236,178 individuals) was downloaded from the FinnGen consortium (R8 data release) (https://www.finngen.fi/en). The definition of PPD in FinnGen was participants with delivery history diagnosed with F32, F33, or F530 in the 10th edition of the International Classification of Diseases criteria.

#### 2.3.4 Selection criteria of genetic instruments

All summary statistics were filtered at the minimum variant allele (MAF) frequency >0.01. SNPs were all selected at the genome-wide significance level (*P* < 5E−8). If there were no or less than four SNPs that met the criteria, a more lenient threshold (*P* < 5E−6) would be applied. Linkage disequilibrium (LD) for each trait was estimated based on the 1000 Genomes LD reference panel in European ancestry with the threshold set to *r* ^2^ >0.01 and clump window of 5,000 kb. SNPs identified as linkage disequilibrium, palindromic, or incompatible were excluded. To verify the third hypothesis of MR, SNPs significantly associated with PPD (*P* < 5E−8) were excluded.

### 2.4 Statistical analysis

#### 2.4.1 Statistical procedure of the observational study

To assess the unadjusted association between PPD and all the other variables, categorical variables were subjected to the chi-square test, and Fisher’s exact test was utilized for variables with small-sample sizes. Continuous variables were analyzed using Student’s *t*-test, while the Wilcoxon–Mann–Whitney test was employed for non-normally distributed variables. To remove variables not associated with PPD, univariate logistic regression analysis was performed on all variables. The odds ratio (OR) and the corresponding 95% confidential interval (CI) were applied to determine the significance. The cutoff *P*-value for univariate logistic regression was 0.1. To further select candidate risk factors connected to PPD, the random forest algorithm on the significant variables selected by the univariate logistic regression was performed with 1,000 random permutations. After the screening process, a multivariate logistic regression analysis was performed to illustrate the independent risk factors.

#### 2.4.2 Bidirectional Mendelian randomization analyses

We designed a bidirectional Mendelian randomization study to determine the causal relationship between bilirubin and PPD. We utilized the inverse variance-weighted (IVW) method as the primary approach for causal inference, which provides a weighted regression of IV-specific causal estimates and a stable causal inference even in the presence of heterogeneity (38). Multivariate Mendelian randomization (MVMR) analyses were performed to adjust potential confounders and explore the direct effect of each variable on the outcome (39). Least absolute shrinkage and selection operator (LASSO) regression was utilized to avoid potential bias caused by multicollinearity.

#### 2.4.3 Sensitivity analyses for Mendelian randomization

To evaluate the reliability of the causal inference between bilirubin and PPD, sensitivity analyses were conducted, comprising weighted median, MR-Egger regression, Cochran’s *Q* test, and MR-PRESSO (pleiotropy residual sum and outlier). The weighted median model can generate consistent estimates, when more than half of the analytical weights are derived from valid IVs (38). MR-Egger regression allows pleiotropy present in more than half of IVs, whereas it compromises statistical power (40). MR-PRESSO is able to detect and correct the bias caused by horizontal pleiotropic outliers (41). Causal inference can only be made when the same direction was reported in IVW estimation, weighted median method, and MR-Egger regression, and the MR-Egger regression intercept test does not detect horizontal pleiotropy. To estimate heterogeneity among SNPs for exposure and assess the consistency between assumption and MR analyses, Cochran’s *Q* test was performed. To evaluate the strength of IVs for exposures in the MR analyses, *F*-statistics were calculated (42, 43). *F*-statistic >10 suggested a strong instrumental variable. All statistical analyses in the present study were performed in R 4.1.0 (https://www.R-project.org/). Logistics regressions were implemented with the R package “rms.” The random forest algorithm was performed by Python 3.10, and all algorithms are available in the python library sklearn (44). The R packages “TwoSampleMR” (45) and “MRPRESSO” (41) were used to perform MR and sensitivity analyses. A two-sided significance level was set as *P*-value <0.05 for all statistical testing. In the figures, the asterisk(s) indicated the following: *, *P* < 0.05; **, *P* < 0.01; ***, *P* < 0.001; and ****, *P* < 0.0001.

### 2.5 Ethics approval

This study was reviewed by the Ethics Committee of West China Second University Hospital, Sichuan University (No. 2021-186) and conducted following the principles of the Declaration of Helsinki. Informed consent was obtained from each participant. All studies included in the GWAS cited in this study were approved by a relevant review board.

## 3 Results

### 3.1 Baseline characteristics of observational studies

A total of 1,810 patients were finally enrolled in this study. As shown in **Table 1**, approximately one-third of the patients suffered from PPD. As for the distribution of total bilirubin level, only 22 individuals reached the high–normal total bilirubin of 23 μmol/L. Uncorrected test results indicated that there were significant differences in the levels of total bilirubin (9.2 μmol/L, IQR 7.7, 11.0 in PPD; 9.7 μmol/L, IQR 8.0, 12.0 in control, *P* < 0.001) and direct bilirubin (2.0 μmol/L, IQR 1.6, 2.6 in PPD; 2.2 μmol/L, IQR 1.7, 2.9 in control, *P* < 0.003) in the serum between PPD patients and the control group. Gestational diabetes was also found significantly different between PPD patients and the control group [155 (24.6%) in PPD; 211 (17.9%) in control, *P* < 0.001]. As for sociodemographic traits, median age (32, IQR 29, 35 in PPD; 31, IQR 29, 34 in control, *P* = 0.003), education [225 (35.7%) below bachelor’s degree in PPD; 331 (28.1%) below bachelor’s degree in control, *P* = 0.001], income [62 (9.8%) low income in PPD; 68 (5.8%) low income in control, *P* = 0.002], work status [615 (97.5%) employed in PPD; 1,119 (94.9%) employed in control, *P* = 0.014], and poor marriage [617 (97.8%) in PPD; 1,170 (99.2%) in control, *P* = 0.016] were statistically different.

**Table 1:** Characteristics of PPD cohort.

Characteristic	Level	Overall	Postpartum depression		P value
			Yes	No	
N (%)		1,810 (100%)	631 (34.9%)	1,179 (65.1%)	
*Demographic information*					
Age, median (IQR)		31 (29, 35)	32 (29, 35)	31 (29, 34)	0.003
Gain weight (kg), median (IQR)		12.5 (9.5, 15.0)	12.5 (9.5, 16.0)	12.5 (9.5, 15.0)	0.605
BMI (kg/m^2^), median (IQR)		20.8 (19.34, 22.86)	20.83 (19.34, 23.05)	20.83 (19.34, 22.77)	0.482
Gestation days, median (IQR)		274 (269, 279)	274 (268, 278)	274 (269, 280)	0.022
Nationality, n (%)	Han	1,757 (97.1%)	608 (96.4%)	1,149 (97.5%)	0.239
	Others	53 (2.9%)	23 (3.6%)	30 (2.5%)	
Season, n (%)	Autumn	560 (30.9%)	197 (31.2%)	363 (30.8%)	0.953
	Spring	335 (18.5%)	118 (18.7%)	217 (18.4%)	
	Summer	465 (25.7%)	157 (24.9%)	308 (26.1%)	
	Winter	450 (24.9%)	159 (25.2%)	291 (24.7%)	
*Sociological information*					
Work status, n (%)	Employed	1,734 (95.8%)	615 (97.5%)	1,119 (94.9%)	0.014
	Unemployed or retired	76 (4.2%)	16 (2.5%)	60 (5.1%)	
Education, n (%)	Bachelor's degree or above	1,254 (69.3%)	406 (64.3%)	848 (71.9%)	0.001
	Below bachelor's degree	556 (30.7%)	225 (35.7%)	331 (28.1%)	
Income, n (%)	Normal level	1,680 (92.8%)	569 (90.2%)	1,111 (94.2%)	0.002
	Low income	130 (7.2%)	62 (9.8%)	68 (5.8%)	
Planning pregnancy, n (%)	No	72 (4.0%)	30 (4.8%)	42 (3.6%)	0.267
	Yes	1,738 (96.0%)	601 (95.2%)	1,137 (96.4%)	
Social support, n (%)	No	42 (2.3%)	18 (2.9%)	24 (2.0%)	0.349
	Yes	1,768 (97.7%)	613 (97.1%)	1,155 (98.0%)	
Poor marriage, n (%)	No	1,787 (98.7%)	617 (97.8%)	1,170 (99.2%)	0.016
	Yes	23 (1.3%)	14 (2.2%)	9 (0.8%)	
*Clinical characteristics*					
Baby weight (kg), median (IQR)		3.26 (2.92, 3.56)	3.23 (2.80, 3.54)	3.28 (2.96, 3.58)	0.004
Baby height (cm), median (IQR)		50 (48, 51)	49 (48, 51)	50 (48, 51)	0.042
Length of stay (day), median (IQR)		4 (4, 6)	4 (4, 6)	4 (4, 6)	0.654
Postpartum hemorrhage (mL), median (IQR)		400 (300, 400)	400 (300, 400)	400 (300, 400)	0.382
Fetal malformation, n (%)	No	1,724 (95.2%)	592 (93.8%)	1,132 (96.0%)	0.048
	Yes	86 (4.8%)	39 (6.2%)	47 (4.0%)	
Gestational diabetes mellitus, n (%)	No	1,444 (79.8%)	476 (75.4%)	968 (82.1%)	< 0.001
	Yes	366 (20.2%)	155 (24.6%)	211 (17.9%)	
Hypertension, n (%)	No	1,725 (95.3%)	604 (95.7%)	1,121 (95.1%)	0.619
	Yes	85 (4.7%)	27 (4.3%)	58 (4.9%)	
Hepatitis B, n (%)	No	1,708 (94.4%)	593 (94.0%)	1,115 (94.6%)	0.678
	Yes	102 (5.6%)	38 (6.0%)	64 (5.4%)	
Twin pregnancies, n (%)	No	1,642 (90.7%)	561 (88.9%)	1081 (91.7%)	0.063
	Yes	168 (9.3%)	70 (11.1%)	98 (8.3%)	
Placenta previ, n (%)	No	1,715 (94.8%)	598 (94.8%)	1117 (94.7%)	1
	Yes	95 (5.2%)	33 (5.2%)	62 (5.3%)	
Uterine myoma, n (%)	No	1,635 (90.3%)	569 (90.2%)	1,066 (90.4%)	0.935
	Yes	175 (9.7%)	62 (9.8%)	113 (9.6%)	
Ovarian cyst, n (%)	No	1,800 (99.4%)	627 (99.4%)	1,173 (99.5%)	0.746
	Yes	10 (0.6%)	4 (0.6%)	6 (0.5%)	
Fetal growth restriction, n (%)	No	1,777 (98.2%)	617 (97.8%)	1,160 (98.4%)	0.462
	Yes	33 (1.8%)	14 (2.2%)	19 (1.6%)	
Preterm birth, n (%)	No	1,575 (87.0%)	522 (82.7%)	1,053 (89.3%)	< 0.001
	Yes	235 (13.0%)	109 (17.3%)	126 (10.7%)	
Delivery mode, n (%)	Cesarean section	679 (37.5%)	249 (39.5%)	430 (36.5%)	0.337
	midwifery	10 (0.6%)	2 (0.3%)	8 (0.7%)	
	Natural birth	1,121 (61.9%)	380 (60.2%)	741 (62.8%)	
Fetal gender, n (%)	Female	925 (51.1%)	321 (50.9%)	604 (51.2%)	0.924
	Male	885 (48.9%)	310 (49.1%)	575 (48.8%)	
Fetal distress, n (%)	No	1,759 (97.2%)	601 (95.2%)	1,158 (98.2%)	< 0.001
	Yes	51 (2.8%)	30 (4.8%)	21 (1.8%)	
Breastfeeding, n (%)	No	28 (1.5%)	17 (2.7%)	11 (0.9%)	0.007
	Yes	1,782 (98.5%)	614 (97.3%)	1,168 (99.1%)	
Pregnancy, n (%)	1	621 (34.3%)	186 (29.5%)	435 (36.9%)	0.014
	2	517 (28.6%)	186 (29.5%)	331 (28.1%)	
	3	358 (19.8%)	132 (20.9%)	226 (19.2%)	
	4	189 (10.4%)	73 (11.6%)	116 (9.8%)	
	>=5	125 (6.9%)	54 (8.6%)	71 (6.0%)	
Abortion, n (%)	0	834 (46.1%)	260 (41.2%)	574 (48.7%)	0.004
	1	545 (30.1%)	194 (30.7%)	351 (29.8%)	
	2	271 (15.0%)	107 (17.0%)	164 (13.9%)	
	>=3	160 (8.8%)	70 (11.1%)	90 (7.6%)	
Parity, n (%)	0	1,130 (62.4%)	388 (61.5%)	742 (62.9%)	0.484
	1	645 (35.6%)	227 (36%)	418 (35.5%)	
	2	33 (1.8%)	15 (2.4%)	18 (1.5%)	
	>=3	2 (0.1%)	1 (0.2%)	1 (0.1%)	
Apgar 10 min, n (%)	0~3	36 (2.0%)	30 (4.8%)	6 (0.5%)	< 0.001
	4~7	5 (0.3%)	3 (0.5%)	2 (0.2%)	
	8~10	1,769 (97.7%)	598 (94.8%)	1,171 (99.3%)	
*Laboratory indicators*					
White blood cell (10^9/L), median (IQR)		9.2 (7.8, 11.1)	9.2 (7.9, 11.0)	9.2 (7.8, 11.1)	0.584
Platelet (10^9/L), median (IQR)		179 (145, 213)	182 (146, 218)	177 (144, 211)	0.125
Hemoglobin (g/L), median (IQR)		112 (104, 118)	111 (104, 118)	112 (104, 117)	0.885
Ferroprotein (ng/nl), median (IQR)		18.4 (12.2, 26.0)	19.0 (11.9, 26.8)	18.1 (12.4, 25.9)	0.518
Prothrombin time (s), median (IQR)		10.8 (10.3, 11.4)	10.8 (10.4, 11.4)	10.8 (10.3, 11.4)	0.817
International normalized ratio, median (IQR)		0.96 (0.92, 1.01)	0.96 (0.91, 1.01)	0.97 (0.92, 1.01)	0.233
Activated partial thromboplastin time, median (IQR)		26.1 (24.8, 27.6)	25.9 (24.8, 27.4)	26.2 (24.8, 27.7)	0.23
Fibrinogen (mg/dL), median (IQR)		416 (356, 470)	416 (355, 470)	414 (358, 470)	0.871
Thrombin time (s), median (IQR)		16.3 (15.8, 16.9)	16.3 (15.8, 16.9)	16.3 (15.8, 16.9)	0.741
Alanine aminotransferase (U/L), median (IQR)		17 (12, 28)	18 (12, 28)	17 (13, 28)	0.927
Aspartate aminotransferase (U/L), median (IQR)		21 (18, 27)	21 (17, 27)	21 (18, 27)	0.627
Total bilirubin (umol/L), median (IQR)		9.5 (7.8, 11.8)	9.2 (7.7, 11.0)	9.7 (8.0, 12.0)	< 0.001
Direct Bilirubin (umol/L), median (IQR)		2.1 (1.7, 2.8)	2.0 (1.6, 2.6)	2.2 (1.7, 2.9)	0.003
Total protein (g/L), median (IQR)		66.0 (62.8, 69.7)	65.7 (62.8, 69.5)	66.2 (62.8, 69.8)	0.244
Albumin (g/L), median (IQR)		38.7 (36.3, 41.3)	38.6 (36.3, 41.4)	38.8 (36.3, 41.2)	0.91
Globulin (g/L), median (IQR)		27.4 (25.2, 30.1)	27.2 (25.2, 29.9)	27.5 (25.4, 30.2)	0.165
Albumin/Globulin, median (IQR)		1.4 (1.3, 1.6)	1.4 (1.3, 1.6)	1.4 (1.3, 1.6)	0.234
R-glutamyltransferase (U/L), median (IQR)		15 (10, 23)	14 (10, 23)	15 (10, 23)	0.816
Lactate dehydrogenase (U/L), median (IQR)		180 (163, 202)	180 (164, 202)	179 (163, 202)	0.894
Alkaline phosphatase (U/L), median (IQR)		85 (55, 122)	87 (55, 124)	84 (55, 121)	0.624
Total bile acid (umol/L), median (IQR)		2.3 (1.6, 3.6)	2.4 (1.6, 3.6)	2.3 (1.6, 3.5)	0.521
Urea nitrogen (umol/L), median (IQR)		3.5 (3.08, 4.35)	3.48 (3.08, 4.35)	3.5 (3.08, 4.35)	0.638
Creatinine (umol/L), median (IQR)		44 (40, 48)	44 (40, 48)	44 (40, 48)	0.732
Cystatin C (mg/L), median (IQR)		0.77 (0.64, 0.98)	0.77 (0.64, 0.99)	0.77 (0.64, 0.97)	0.751
Uric acid (umol/L), median (IQR)		257 (217, 307)	254 (218, 304)	259 (217, 309)	0.265
Thyroid stimulating hormone (mIU/L), median (IQR)		1.95 (1.25, 2.86)	1.86 (1.17, 2.79)	1.97 (1.30, 2.89)	0.166
FT4 (pmol/L), median (IQR)		14.54 (13.22, 16.08)	14.53 (13.21, 15.88)	14.55 (13.27, 16.18)	0.397
Thyroid peroxidase antibody (U/ml), median (IQR)		41.2 (30.4, 56.1)	41.1 (30.7, 55.6)	41.2 (30.4, 56.2)	0.541

PPD postpartum depression, IQR interquartile range, BMI body mass index.

In contrast to prior studies, it appears that the mode of delivery was not significantly associated with PPD (*P* = 0.337), and neither was thyroid function (TSH, *P* = 0.166; FT4, *P* = 0.397).

### 3.2 Identification of risk factors of postpartum depression

**Figure 2:**
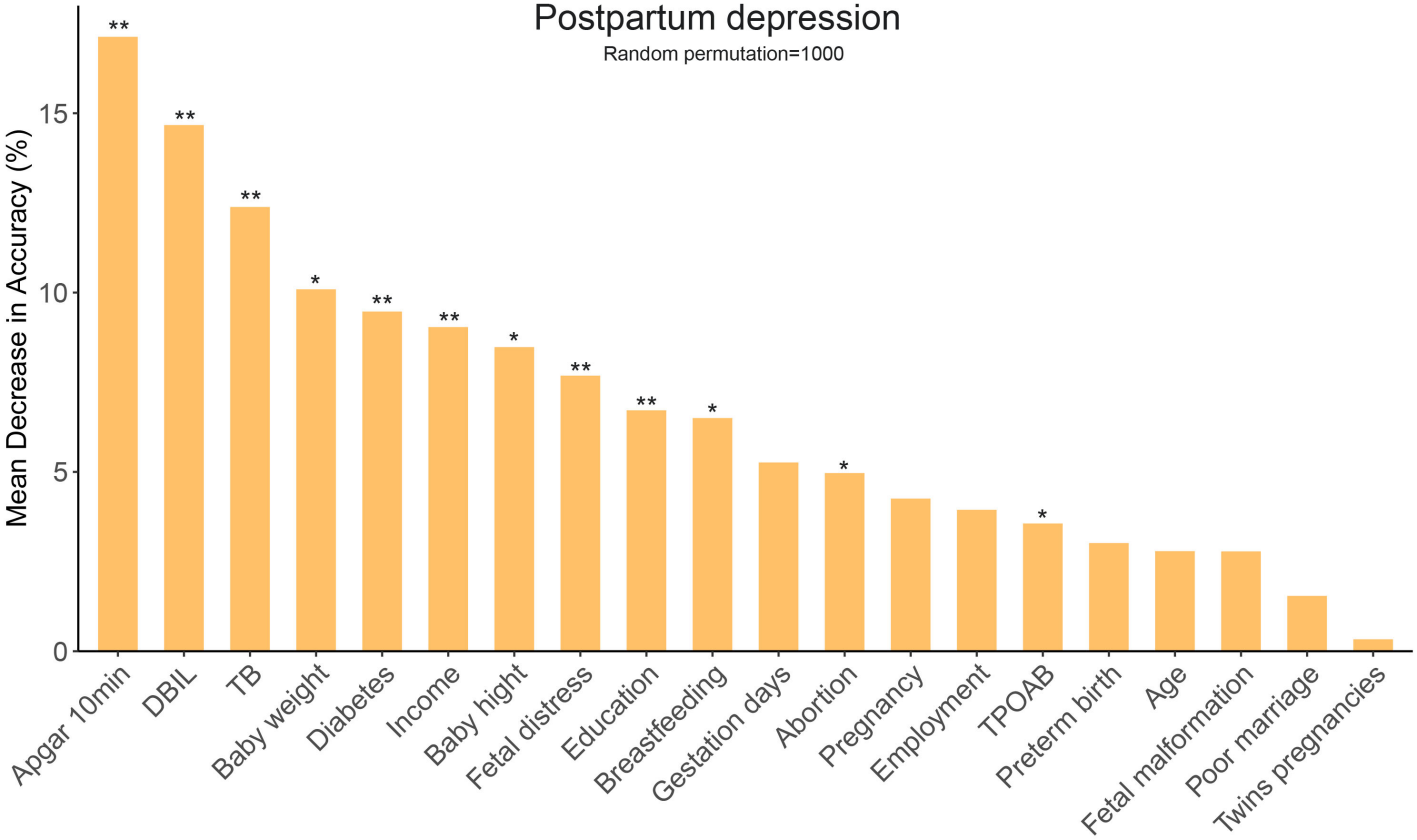
Random forest algorithm for selecting important variables related to PPD. PPD, postpartum depression; TB, total bilirubin; DBIL, direct bilirubin; TPOAB, thyroid peroxidase antibody. *, P < 0.05; **, P < 0.01.

To rule out the irrelevant variables of PPD, univariate logistic regression analysis was conducted on all 62 variables. Of these, 20 were found to be statistically significant (*P* < 0.1, **Table 2**). The random forest algorithm was further performed and identified 12 important variables associated with PPD (**Figure 2**). Next, multivariate logistic regression analysis was conducted to adjust confounding factors of PPD. Finally, as presented in **Table 2**, Apgar score at 10 min (OR = 0.79, 95% CI 0.71–0.88, *P* < 0.001), the serum level of total bilirubin (OR = 0.94, 95% CI 0.90–0.99, *P* = 0.024), thyroid peroxidase antibodies (TPOAbs) (OR = 1.00, 95% CI 0.99–1.00, *P* = 0.002), bachelor’s degree or above (OR = 0.73, 95% CI 0.59–0.92, *P* = 0.006), and breastfeeding (OR = 0.39, 95% CI 0.17–0.86, *P* = 0.02) were identified as independent protective factors against PPD. Conversely, low income (OR = 1.54, 95% CI 1.05–2.26, *P* = 0.025), gestational diabetes mellitus (OR = 1.69, 95% CI 1.32–2.16, *P* < 0.001), and the number of abortions were associated with an increased risk of PPD.

**Table 2:** Univariate and multivariate logistic regression of variables of PPD.

Characteristics	Univariate		Multivariate	
	OR (95% CI)	P value	OR (95% CI)	P value
Age	1.04 (1.01, 1.06)	0.002		
Gestation days	0.99 (0.98, 0.99)	<0.001		
Baby weight	0.70 (0.60, 0.82)	<0.001	0.72 (0.51 - 1.01)	0.058
Baby height	0.94 (0.91 0.97)	<0.001	1.06 (0.98 - 1.14)	0.136
Apgar 10min	0.80 (0.73, 0.87)	<0.001	0.79 (0.71 - 0.88)	<0.001
Total bilirubin	0.94 (0.91, 0.97)	<0.001	0.94 (0.90 - 0.99)	0.024
Direct bilirubin	0.85 (0.77, 0.92)	<0.001	0.95 (0.82 - 1.08)	0.447
TPOAB	1.00 (0.99, 1.00)	0.002	1.00 (0.99 – 1.00)	0.002
Work status				
Employed	Reference			
Unemployed	0.49 (0.27, 0.83)	0.011		
Education				
Below bachelor's degree	Reference		Reference	
Bachelor’s degree or above	0.70 (0.57, 0.87)	0.001	0.73 (0.59 - 0.92)	0.006
Income				
Normal level	Reference		Reference	
Low income	1.78 (1.24, 2.55)	0.002	1.54 (1.05 - 2.26)	0.025
Poor marriage				
No	Reference			
Yes	2.95 (1.29, 7.12)	0.012		
Fetal malformation				
No	Reference			
Yes	1.59 (1.02, 2.45)	0.038		
Diabetes				
No	Reference		Reference	
Yes	1.49 (1.18, 1.89)	0.001	1.69 (1.32 - 2.16)	<0.001
Twin pregnancies				
No	Reference			
Yes	1.38 (0.99, 1.90)	0.053		
Preterm birth				
No	Reference			
Yes	1.75 (1.32, 2.30)	<0.001		
Fetal distress				
No	Reference			
Yes	2.75 (1.57, 4.91)	<0.001		
Breastfeeding				
No	Reference		Reference	
Yes	0.34 (0.15, 0.72)	0.006	0.39 (0.17 - 0.86)	0.02
Number of pregnancies				
1	Reference			
2	1.31 (1.03, 1.69)	0.031		
3	1.37 (1.04, 1.80)	0.026		
4	1.47 (1.05, 2.06)	0.026		
>=5	1.78 (1.20, 2.63)	0.004		
Number of abortions				
0	Reference		Reference	
1	1.22 (0.97, 1.53)	0.088	1.20 (0.95 - 1.52)	0.128
2	1.44 (1.08, 1.91)	0.012	1.38 (1.03 - 1.86)	0.032
>=3	1.72 (1.21, 2.42)	0.002	1.72 (1.20 - 2.47)	0.003

PPD postpartum depression, OR odds ratio, CI confidence interval, TPOAB thyroid peroxidase antibody.

### 3.3 Selection and verification of instrumental variables

For serum bilirubin, 122 and 63 independent SNPs were selected as IVs for total and direct bilirubin (**Supplementary Tables 1**, **2**) at the genome-wide significance level (*P* < 5E−8), respectively. Eight palindromic SNPs for total bilirubin and six palindromic SNPs for direct bilirubin were excluded to ensure the harmonization of the effect of IVs on the outcome and exposure. For PPD, a less stringent significance threshold (*P* < 5E−6) was used in IV selection (**Supplementary Tables 3**, **4**). One palindromic SNP was deleted. All *F*-statistics of IVs were above 10, suggesting strong IVs.

### 3.4 The causal effects of bilirubin on postpartum depression

**Figure 3:**
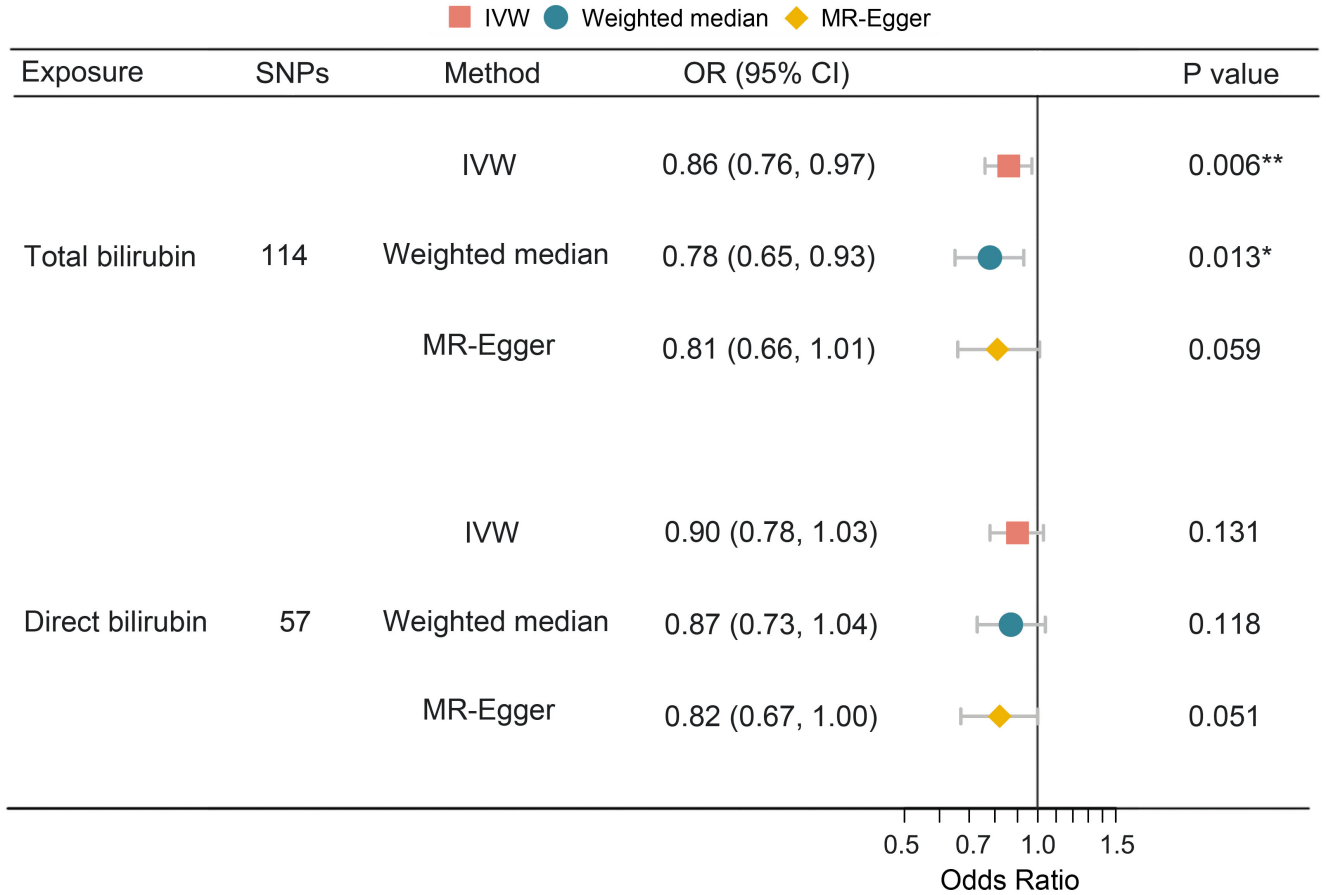
Univariate MR analysis of the causal effect of bilirubin on PPD. MR, Mendelian randomization; PPD, postpartum depression; SNP, single nucleotide polymorphism; OR, odds ratio; CI, confidence interval; IVW, inverse variance weighting. *, P < 0.05; **, P < 0.01.

To investigate the causality of bilirubin on PPD, univariate MR analysis was performed using total and direct bilirubin as exposure, respectively. The results showed that an increased level of serum total bilirubin was significantly associated with a decreased risk of PPD (IVW: OR = 0.86, 95% CI 0.76–0.97, *P* = 0.006), while the causal association for direct bilirubin was not statistically significant (IVW: OR = 0.90, 95% CI 0.78–1.03, *P* = 0.131) (**Figure 3**).

**Figure 4:**
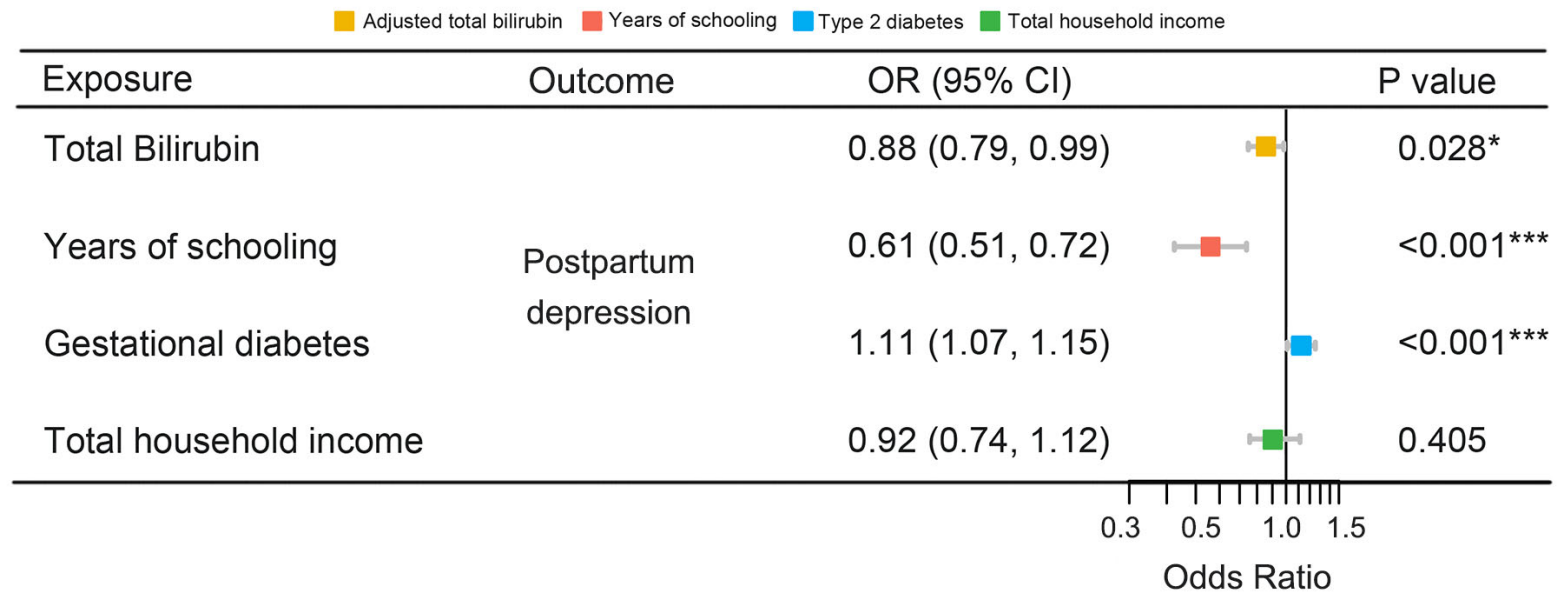
MVMR of the causal effect of bilirubin on PPD. MVMR, multivariable Mendelian randomization; PPD, postpartum depression; OR, odds ratio; CI, confidence interval. *, P < 0.05; ***, P < 0.001.

**Table 3** illustrates the results of the sensitivity analyses. Consistent directions of the causal estimates were observed in the weighted median and MR-Egger methods. No significant outlier was detected by the MR-PRESSO outlier test. No significant evidence of horizontal pleiotropy was observed for total (*P* = 0.576) and direct (*P* = 0.195) bilirubin. However, Cochran’s *Q* test reported mild heterogeneity in both the SNPs of total bilirubin (*P* = 0.010) and direct (*P* = 0.026) bilirubin. To validate the direct causal effects of bilirubin on PPD, MVMR was carried out. Total bilirubin, direct bilirubin, year of schooling, average total household income before tax, and gestational diabetes mellitus were admitted as exposures. Due to potential multicollinearity between exposures, direct bilirubin was excluded from the MVMR analysis via LASSO regression variable selection. As shown in **Figure 4**, the result for total bilirubin remained consistent after adjusting educational attainment (years of schooling), income (average total household income before tax), and gestational diabetes mellitus. Interestingly, higher educational attainment was demonstrated to be a protective factor of PPD (OR = 0.61, 95% CI 0.51–0.72, *P* < 0.001), while gestational diabetes mellitus was hazardous (OR = 1.11, 95% CI 1.07–1.15, *P* < 0.001), which were consistent with the cohort observation and prediction model. Taken together, these results suggested a genuine causality of total bilirubin on PPD after adjusting educational attainment, income, and gestational diabetes mellitus.

**Table 3:** Sensitivity analysis of causality between bilirubin and postpartum depression.

Exposure	Outcome	Weighted median		MR-Egger regression		Heterogeneity^a^	MR-PRESSO outlier detect^b^		Pleiotropy^c^
		OR/Beta (95% CI)	P Value	OR/Beta (95% CI)	P Value		OR (95% CI)	P Value	
TB	PPD	0.779 (0.653, 0.930)	0.006	0.814 (0.659, 1.005)	0.059	I^2^ = 25.2%; Cochrane's Q = 151; P_het_ =0.010	No significant outliers		Intercept = 0.002; P_ple_ = 0.576
DBIL	PPD	0.869 (0.728, 1.036)	0.118	0.815 (0.667, 0.996)	0.051	I^2^ = 28.6%; Cochrane's Q = 77; P_het_ = 0.026	No significant outliers		Intercept = 0.005; P_ple_ = 0.195
PPD	TB	-0.020 (-0.041, -0.010)	0.083	0.052 (-0.030, 0.131)	0.230	I2 = 16.7%; Cochrane's Q = 12; het = 0.285	No significant outliers		Intercept = -0.008; Pple = 0.092
PPD	DBIL	-0.023 (-0.041, 0.020)	0.071	0.024 (-0.062, 0.113)	0.593	I2 = 0.1%; Cochrane's Q = 10; Phet = 0.470	No significant outliers		Intercept = -0.005; Pple = 0.320

a Heterogeneity in the random effect IVW methods was reported. Mild heterogeneity was observed in both the SNPs of total bilirubin and direct bilirubin.

b There is no outlier needed to be corrected.

c MR-Egger was used to detect Pleiotropy. No pleiotropy was observed (P>0.05).

PPD postpartum depression, TB total bilirubin, DBIL direct bilirubin, OR odds ratio, CI confidence interval, MR mendelian randomization.

### 3.5 Reverse causal effects of postpartum depression on bilirubin

**Figure 5:**
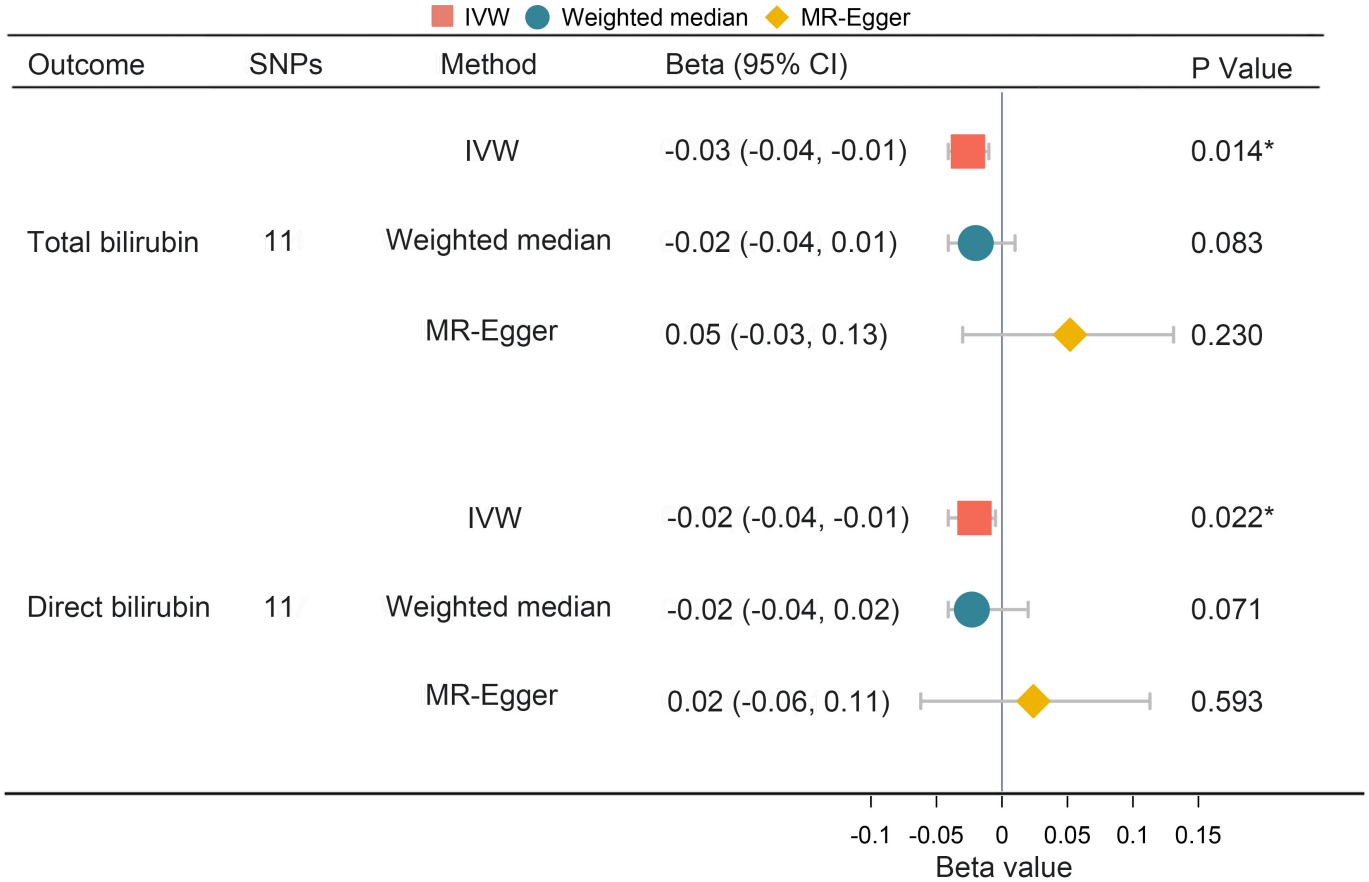
Univariate MR analysis of the causal effect of PPD on bilirubin. MR, Mendelian randomization; PPD, postpartum depression; SNP, single nucleotide polymorphism; OR, odds ratio; CI, confidence interval; IVW, inverse variance weighting. *, P < 0.05.

To investigate the directionality of the causal relationship between bilirubin and PPD, we further conducted reverse MR analyses. The results demonstrated that PPD showed statistically significant causal effects on both total and direct bilirubin (IVW: Beta = -0.03, 95% CI -0.04, -0.01, *P* = 0.014 and IVW: Beta = -0.02, 95% CI -0.04, -0.01, *P* = 0.022 respectively) as shown in **Figure 5**. However, MR-Egger regression described an opposite direction (total bilirubin Beta = 0.052, 95% CI -0.030, 0.131, P = 0.230, *P* = 0.23; direct bilirubin Beta = 0.024, 95% CI -0.062, 0.113, *P* = 0.593) (**Figure 5**). No evidence of horizontal pleiotropy or heterogeneity was detected. The MR-PRESSO outlier test did not identify any significant outliers (**Table 3**). Collectively, there is a lack of concrete results to support the causal inference from PPD on neither total bilirubin nor direct bilirubin.

## 4 Discussion

As a common psychological disease, PPD has a severe influence on the family and society and requires appropriate management (6). The pathology of PPD is not yet clear, although dozens of socioeconomic, psychological, and physiological predictors were proposed and verified by clinical studies (8). Combining observational and Mendelian randomization study, we described the association and causality between bilirubin and PPD for the first time.

In our observational cohort, a decreased serum level of total bilirubin was associated with an increased risk of PPD, while the serum level of direct bilirubin was not. Meanwhile, bidirectional two-sample MR and MVMR with published GWAS data confirmed the true causality of total bilirubin on PPD. On the other hand, the occurrence of PPD resulting in a decrease in serum bilirubin should be modestly interpreted considering the ambiguous outcomes of reverse MR. As secondary results, gestational diabetes mellitus and lower educational attainment were associated with an increased risk of PPD.

For a very long period of time, bilirubin has been considered a waste product of heme catabolism with neurotoxicity, and serum bilirubin level has been used as an ominous sign of liver disease, until Stocker et al. reported the antioxidant capacity of bilirubin in 1987 (14). Heme, one of the decomposed products of erythrocytes, was catabolized to produce biliverdin, which is reduced to indirect bilirubin, also known as unconjugated bilirubin. Then, unconjugated bilirubin is released into the circulation and is combined with albumin before entering the hepatocyte, where it is conjugated with glucuronic acid to form conjugated bilirubin or direct bilirubin. After that, direct bilirubin is excreted by the hepatocyte into the biliary tract, and a disorder of bilirubin excretion would result in elevated direct bilirubin in the serum, which is called obstructive jaundice (46).

Oxidative stress (OS), defined as the imbalance between the production of reactive oxygen species (ROS) and endogenous antioxidants, was widely considered as one of the major hypotheses of the pathogenesis of depression (47). ROS overload could induce the expression of heme oxygenase (HO-1), which catalyzes the decomposition of heme. As a result, increased bilirubin is able to reduce ROS via redox (48). OS has been proposed to be one of the major causes of depressive disorder, and representative biomarkers of OS were increased in patients with depression (49). Similar results were reported in PPD in recent studies, indicating the elevated status of OS in PPD patients (50). Urinary biopyrrin, the production of the oxidation reaction of bilirubin with reactive oxygen, has been used as an oxidative stress marker (51). A higher level of urinary biopyrrin was found in patients with depression and schizophrenia, which suggested more consumption of bilirubin caused by OS in psychiatric disorders (52).

Based on the aforementioned studies and the results of the present research, decreased bilirubin might impair the antioxidant defense system, inducing oxidative damage in pregnant women, which could possibly lead to PPD. As ROS mainly causes damage inside the cells where direct bilirubin is difficult to reach, the true antioxidant is indirect bilirubin, which accounts for most of the total circulation bilirubin under physiological conditions (47). The level of bilirubin could increase due to liver dysfunction, which occurred in up to 3% of pregnant women (53). Decreased total and direct bilirubin levels were usually due to anemia. In Japanese participants with self-reported depression, a higher rate of self-reported history of iron deficiency anemia was observed, which was in accordance with our theory (54). The relationship between indirect bilirubin and PPD should be further investigated.

In the previous MR research, a significant causal effect of major depressive disorders on total bilirubin was reported, which implied a sophisticated relationship between depression and bilirubin (25). Given that most of the serum levels of bilirubin of participants in the observational study and MR analysis were within normal limits, the acute neurotoxic effect of high-level bilirubin had little impact on our research. In poststroke and diabetes patients who also suffered from depression, elevated bilirubin was observed (16–18). This opposite association was not equal to true causality; instead, bilirubin might function as a protector against depression in the pathologic state. Further research might provide more clues on this matter. Recently, multidimensional evidence suggested that bilirubin was more than just an antioxidant; it also might serve as a messenger of cell signal transduction, metabolism modulation, and immune regulation (55). Mechanisms other than OS might be involved in the pathogenesis of PPD.

There were several highlights in our research. First, our PPD cohort was built with the standard protocol of medical care based on evidence-based clinical guidelines, and a quality control of follow-up was executed. Second, the major results of the observational study were confirmed by MR analysis, which minimized the confounding effect and reverse causality. Moreover, no pleiotropic effect was detected in the sensitivity analyses, which indicated that causal estimates were not induced by confounders. Finally, consistent positive results were observed in both the Asian cohort (observational study) and the European cohort (MR study), suggesting that the causal association of bilirubin with PPD is robust and generalizable.

On the other hand, limitations should be declared equally. First, two different diagnostic criteria of PPD were applied in our cohort and the FinnGen database, and the criteria in our cohort lacked the diagnosis of depression by a psychiatrist after childbirth. Second, participants with incomplete results of perinatal examinations were excluded from the observational study, which might be a source of selection bias. Third, there was a potential overlap in GWAS for PPD and gestational diabetes mellitus in MVMR, which might cause fake positive results. Furthermore, mild heterogeneity was observed in the SNPs of both total and direct bilirubin. However, the use of the random-effect IVW method and the absence of horizontal pleiotropy suggested that our results were unlikely to be disturbed by heterogeneity. Fourth, the observational cohort only included participants who were within the normal levels of bilirubin; thus, whether the abnormally increased bilirubin is protective for depression in postpartum women remained to be discovered. Lastly, while the causal relationship of bilirubin on PPD was hinted at by a clinical cohort and further validated by MR analysis, many potential unadjusted confounders in the MR study could be behind this association. A well-designed multicenter prospective PPD cohort was needed to generate credible data.

In a nutshell, our results suggested a clinical association between total bilirubin and PPD, and decreased total bilirubin was likely to be a cause of PPD. Further studies regarding the biological function of bilirubin and its association with PPD are warranted.

## 5 Conclusion

In conclusion, the present research demonstrated that the decreased serum level of total bilirubin was associated with an increased risk of PPD. The importance of serum bilirubin levels on PPD surveillance and prevention should be addressed and further studies are required.

## Data availability statement

The raw data supporting the conclusions of this article will be made available by the authors, without undue reservation. All the cited GWAS data was downloaded from public database.

## Ethics statement

The studies involving humans were approved by the Ethics Committee of West China Second University Hospital, Sichuan University. The studies were conducted in accordance with the local legislation and institutional requirements. The participants provided their written informed consent to participate in this study.

## Author contributions

YL: Writing – original draft, Writing – review & editing. ZW: Writing – original draft, Writing – review & editing. DL: Writing – original draft, Writing – review & editing. BL: Writing – original draft, Writing – review & editing.
